# Alterations in Dynamic Functional Connectivity in Individuals With Subjective Cognitive Decline

**DOI:** 10.3389/fnagi.2021.646017

**Published:** 2021-02-03

**Authors:** Qian Chen, Jiaming Lu, Xin Zhang, Yi Sun, Wenqian Chen, Xin Li, Wen Zhang, Zhao Qing, Bing Zhang

**Affiliations:** ^1^Department of Radiology, Drum Tower Hospital, Clinical College of Nanjing Medical University, Nanjing, China; ^2^Department of Radiology, Drum Tower Hospital, Medical School of Nanjing University, Nanjing, China; ^3^Institute of Brain Science, Nanjing University, Nanjing, China

**Keywords:** subjective cognitive decline, dynamic functional connectivity, independent component analysis, graph theory, fractional windows

## Abstract

**Purpose:** To investigate the dynamic functional connectivity (DFC) and static parameters of graph theory in individuals with subjective cognitive decline (SCD) and the associations of DFC and topological properties with cognitive performance.

**Methods:** Thirty-three control subjects and 32 SCD individuals were enrolled in this study, and neuropsychological evaluations and resting-state functional magnetic resonance imaging scanning were performed. Thirty-three components were selected by group independent component analysis to construct 7 functional networks. Based on the sliding window approach and k-means clustering, distinct DFC states were identified. We calculated the temporal properties of fractional windows in each state, the mean dwell time in each state, and the number of transitions between each pair of DFC states. The global and local static parameters were assessed by graph theory analysis. The differences in DFC and topological metrics, and the associations of the altered neuroimaging measures with cognitive performance were assessed.

**Results:** The whole cohort demonstrated 4 distinct connectivity states. Compared to the control group, the SCD group showed increased fractional windows and an increased mean dwell time in state 4, characterized by hypoconnectivity both within and between networks. The SCD group also showed decreased fractional windows and a decreased mean dwell time in state 2, dominated by hyperconnectivity within and between the auditory, visual and somatomotor networks. The number of transitions between state 1 and state 2, between state 2 and state 3, and between state 2 and state 4 was significantly reduced in the SCD group compared to the control group. No significant differences in global or local topological metrics were observed. The altered DFC properties showed significant correlations with cognitive performance.

**Conclusion:** Our findings indicated DFC network reconfiguration in the SCD stage, which may underlie the early cognitive decline in SCD subjects and serve as sensitive neuroimaging biomarkers for the preclinical detection of individuals with incipient Alzheimer's disease.

## Introduction

Individuals with subjective cognitive decline (SCD), a self-perceived worsening of cognitive function without objectively detected deficits, have been considered at higher risk of developing Alzheimer's disease (AD) dementia in the future compared to those without cognitive complaints (Reisberg et al., 2010; Jessen et al., 2014). AD is a progressive neurodegenerative disorder that has three stages: the preclinical stage, mild cognitive impairment (MCI), and dementia (Sperling et al., 2011). SCD corresponds to the preclinical stage of the AD spectrum and has the potential to be an effective symptomatic indicator for future cognitive impairment (Dubois et al., 2016; López-Sanz et al., 2017). Due to the lack of effective therapeutic methods targeting late-stage AD patients, it is critical to investigate brain alterations in the SCD stage to pave the way for early diagnosis and intervention (Rabin et al., 2017; Jessen et al., 2020).

Resting-state functional magnetic resonance imaging (rs-fMRI), which reflects intrinsic brain activity, has been proven to be an effective and non-invasive approach for exploring the neural mechanisms underlying neurological disorders (Biswal et al., 1995; Lau et al., 2016). More specifically, functional connectivity (FC), which is defined as the temporal correlation of blood oxygenation level-dependent (BOLD) signals between voxels or brain regions, indicates information processing and transference across functionally coordinated brain networks (Fox et al., 2005). Cognitive impairment could be partly attributable to altered functional coupling in brain-wide networks, and previous studies have reported aberrant FC and disrupted brain networks in AD dementia and MCI patients (Delli Pizzi et al., 2019; Franzmeier et al., 2019). Studies conducted in the SCD cohort have also revealed decreased average FC in the posterior memory system and between the retrosplenial cortex and precuneus (Viviano et al., 2019), reduced FC in cortical midline structures (Yasuno et al., 2015), increased FC between the retrosplenial cortex and frontal cortex (Dillen et al., 2016), and increased occipital and parietal FC associated with the severity of memory complaints compared to normal controls (NCs) (Kawagoe et al., 2019). Therefore, altered FC could be the neural basis underlying early cognitive decline and serve as an objective imaging marker to identify preclinically at-risk AD patients.

To date, most aforementioned rs-fMRI studies have focused on static FC (SFC); however, researchers have suggested that the brain is intrinsically a dynamic system with discrete FC patterns switching rapidly during acquisition (Allen et al., 2014; Vidaurre et al., 2017). Thus, the dynamic characteristics of FC provide a novel perspective on the temporal aspects of information processing across brain networks compared to SFC analysis (Peraza et al., 2015; Schumacher et al., 2019). Currently, dynamic FC (DFC) analysis has been proven to be a promising approach for exploring neural substrates for a variety of neuropsychological disorders, including Parkinson's disease (Díez-Cirarda et al., 2018; Fiorenzato et al., 2019), schizophrenia (Damaraju et al., 2014), and AD (Jones et al., 2012; Córdova-Palomera et al., 2017; Demirtaş et al., 2017; Brenner et al., 2018). More specifically, AD dementia patients were suggested to spend less time in brain functional states with strong posterior default mode network (DMN) region contribution and more time in states with greater anterior DMN region contribution compared to NCs (Jones et al., 2012), and show alterations in local DFC within the temporal, frontal-superior and default-mode networks, as well as decreased global metastability between functional states compared to patients with mild or subjective cognitive impairment and NCs (Córdova-Palomera et al., 2017; Demirtaş et al., 2017). In addition, studies have shown that amnestic MCI patients were more likely to reveal a single dominant state and spent greater time in a costly state relative to the most common state, which may be attributable to reduced flexibility in resource allocation (Brenner et al., 2018). Furthermore, studies have revealed higher accuracy using DFC features to distinguish AD dementia or MCI patients from NCs than SFC features (De Vos et al., 2018; Jie et al., 2018). Alterations in functional network dynamics have been suggested to be related to variations in the subclinical range of memory performance, increased iron accumulation, and the genetic risk of AD (Quevenco et al., 2017). However, few studies have investigated DFC characteristics in SCD individuals. A recent DFC study has shown changes in centrality frequency (the proportion of time a hub with a high degree centrality appeared across the entire time window) in the DMN in SCD individuals, the abnormality of which was related to cognitive performance (Xie et al., 2019). Another recent work has observed higher classification accuracies in distinguishing SCD individuals from NCs using temporal flexibility and spatiotemporal diversity, two measures of DFC, than static parameters of graph theory and structural metrics of voxel-based morphometry analysis (Dong et al., 2020). However, studies employing the DFC temporal properties of fractional windows, mean dwell time, and the number of transitions to SCD subjects are still lacking; these features have been commonly described and proven to be associated with cognition, behavior, and clinical variables in other neuropsychological diseases (Kim et al., 2017; Li et al., 2017; Liu et al., 2017; Díez-Cirarda et al., 2018; Fiorenzato et al., 2019).

Graph theory has been widely used in the investigation of topological features of brain functional networks (Watts and Strogatz, 1998). AD is described as a disconnection syndrome, and previous studies have demonstrated disrupted communication in peripheral regions and preserved organization in rich-club regions in SCD participants (Yan et al., 2018). A recent study based on the Alzheimer's Disease Neuroimaging Initiative (ADNI) has observed higher nodal topological properties (nodal strength, nodal global efficiency, and nodal local efficiency) in SCD individuals than in NCs, and the altered graphic parameters were significantly correlated with amyloid-β and memory function, indicating the compensatory mechanism of the functional connectome underlying SCD (Chen et al., 2020). These findings have suggested the vulnerability of network topology in the SCD stage.

In the present study, we aimed to investigate neuroimaging biomarkers in SCD subjects from both dynamic and static rs-fMRI perspectives and to explore whether temporal properties of DFC were more sensitive than static parameters of graph theory in the SCD stage. We also endeavored to determine the relationships between rs-fMRI measures and cognitive performance. Accordingly, we hypothesized that altered DFC temporal properties of fractional windows, mean dwell time, and state transitions would be observed in SCD subjects, which may improve the present understanding of the neural basis underlying early cognitive decline and provide more promising neuroimaging biomarkers for the detection of incipient AD patients than global and local graphic parameters of SFC.

## Methods

### Subjects

The present study included 32 SCD individuals matched for age, gender, and years of education with 33 NCs. All participants were recruited from the Drum Tower district of Nanjing by advertisement. Individuals could participate in this study if they were 55–75 years old, right-handed, and had at least 9 years of education; in contrast, individuals with a history of stroke, other neuropsychiatric disorders (Parkinson's disease, epilepsy, brain tumor, etc.), severe anxiety or depression, and MRI contraindications were excluded. Individuals showing objective impairment in the following cognitive evaluations were also excluded from the present study (Li et al., 2019). Specifically, three cognitive domains each containing two subtests were assessed: auditory verbal learning test (AVLT) long-delayed memory and AVLT recognition for episodic memory; trail making test part A (TMT-A) and part B (TMT-B) for executive function; and Boston naming test (BNT) and animal fluency test (AFT) for language ability. Participants were considered MCI patients if they had scores >1 standard deviation (SD) below the normative means in both subtests within one cognitive domain or >1 SD below the normative means in three single tests in three different domains. Subjects with memory complaints within the last 5 years and expressed worries associated with memory decline were assigned to the SCD group; those without memory complaints and cognitive impairments were recruited as NCs. The study was conducted according to the Declaration of Helsinki and approved by the institutional review boards of the Nanjing Drum Tower Hospital. Written informed consent was acquired from each participant after a detailed introduction of the study procedure involved.

### Neuropsychological Assessment

The standardized cognitive evaluation was performed by an experienced psychologist. The mini-mental state examination (MMSE) was used to assess global cognition. Another five cognitive domains were evaluated: (1) episodic memory measured with the AVLT, including immediate memory, short-delayed memory, long-delayed memory, cued recall, and recognition; (2) executive function tested with the TMT-A and TMT-B; (3) language function evaluated with the BNT and AFT; (4) processing speed tested with the symbol digit modalities test (SDMT); (5) visuospatial ability assessed with the clock drawing test (CDT).

### Image Acquisition

Imaging data were acquired on a 3T Philips Achieva TX MRI scanner using an 8-channel head coil in the Nanjing Drum Tower Hospital. The parameters of rs-fMRI were set as follows: field of view (FOV) = 192 × 192 mm^2^; slice thickness = 4 mm; matrix size = 64 × 64; repetition time (TR) = 2000 ms; echo time (TE) = 30 ms; flip angle = 90°; number of slices = 35; voxel size = 3 × 3 × 4 mm with no gap. In total, 230 volumes were acquired. Participants were instructed to lie quietly with their eyes closed and stay awake during rs-fMRI scanning. The T_1_-weighted images were obtained with the following parameters: TR = 7,600 ms; TE = 3,400 ms; flip angle = 8°; FOV = 256 × 256 × 192 mm^3^ and slice thickness = 1 mm.

### Image Pre-processing

Pre-processing for rs-fMRI data was performed using the Data Processing Assistant for rs-fMRI advanced edition (DPARSFA, vision 4.3, http://www.restfmri.net) (Chao-Gan and Yu-Feng, 2010). Slice timing, realignment, nuisance regression (white matter and cerebrospinal fluid (CSF) signals and Friston 24 head motion parameters), and spatial normalization to standard Montreal Neurological Institute (MNI) space were carried out. Then all images were smoothed with a 6 mm full-width at half-maximum (FWMH) Gaussian kernel. Realignment parameters were checked, and none showed displacement above 3.0 mm or angular rotation higher than 3.0° among included participants. Two-sample *t*-tests indicated no significant differences in the mean framewise displacement (Jenkinson) (Jenkinson et al., 2002) between the NC and SCD groups (0.11 ± 0.06 mm vs. 0.11 ± 0.07 mm, *p* = 0.916).

### Group Independent Component Analysis

After data pre-processing, spatial group independent component analysis (ICA) was conducted to decompose the data into seven functional networks using the Group ICA of fMRI Toolbox (GIFT) (Calhoun et al., 2001a). Two data reduction steps were performed in the principal component analysis (Allen et al., 2014). First, subject-specific data were reduced to 120 principal components and were concatenated across time. Then, the group-level data were decomposed into 100 components with the expectation-maximization algorithm (Roweis, 1998). We repeated the Infomax ICA algorithm in ICASSO 20 times to ensure stability and reliability (Himberg et al., 2004). Subject-specific spatial maps and time courses were extracted by the back-construction approach (GICA) implemented in GIFT software (Calhoun et al., 2001b).

**Figure 1:**
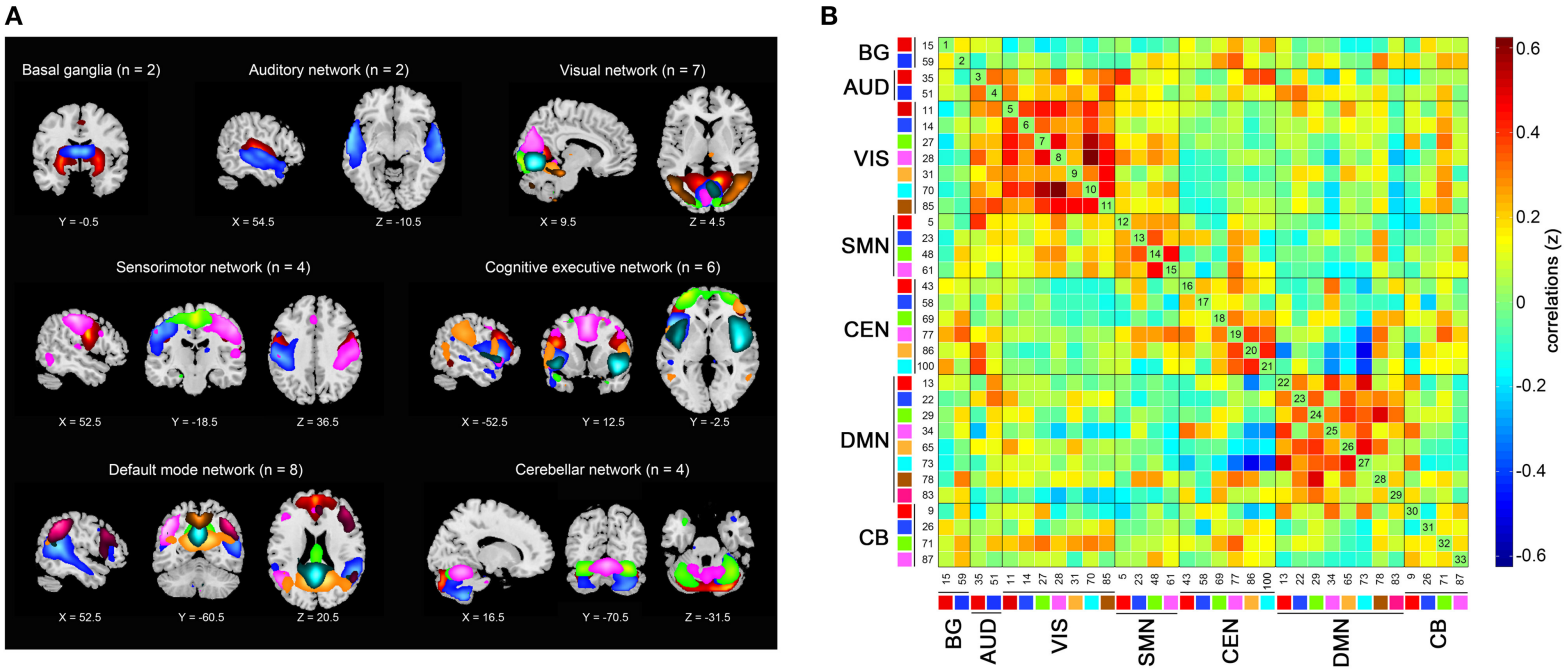
Independent components (*n* = 33) identified by group independent component analysis. **(A)** Independent component spatial maps divided into seven functional networks. **(B)** Group averaged static functional connectivity matrix between pairs of independent components. BG, basal ganglia; AUD, auditory; VIS, visual; SMN, sensorimotor; CEN, cognitive executive; DMN, default mode; CB, cerebellar.

Among the resulting 100 components, we identified 33 of them to construct seven functional networks following a previously described procedure (Allen et al., 2014). First, we manually checked whether the peak activation coordinates were mainly located in gray matter, showing low spatial overlap with vascular, ventricular, or edge regions corresponding to artifacts. Then, only components showing time courses dominated by low-frequency fluctuations were selected (Cordes et al., 2000). Based on the spatial correlation values between the components and the network template (Shirer et al., 2012), we sorted and rearranged the retained 33 independent components into seven functional networks (Figure 1): 2 to the basal ganglia network (BG), 2 to the auditory network (AUD), 7 to the visual network (VIS), 4 to the sensorimotor network (SMN), 6 to the cognitive executive network (CEN), 8 to the DMN, and 4 to the cerebellar network (CB).

Postprocessing steps of the time courses of 33 components were performed according to Allen et al. (2014), including detrending, despiking with AFNI's 3dDespike algorithm, and filtering using a fifth-order Butterworth filter with a 0.15 Hz high frequencies cut-off.

### Dynamic Functional Connectivity Analysis

#### Sliding Window Approach

The DFC analysis was performed with the sliding window approach using the DFC network toolbox in GIFT. Consistent with previous studies, the rs-fMRI data were divided into windows of 22 TR in size with a Gaussian of σ = 3 TRs, in steps of 1 TR (Allen et al., 2014). The regularized inverse covariance matrix was used to reduce the impact of insufficient information on short time series (Varoquaux et al., 2010). We applied an L1 penalty on the precision matrix to promote sparsity in the graphic LASSO framework with 100 repetitions (Friedman et al., 2008). The FC matrices were z-transformed to stabilize the variance.

#### Clustering Analysis and Calculation of Temporal Properties

All windowed FC matrices across all subjects were used to estimate the DFC states. The k-means clustering analysis was repeated 100 times, and the Euclidean distance was used to measure the similarity between FC matrices and regroup them into distinct clusters (Díez-Cirarda et al., 2018). Four was determined as the optimal number of clusters following the elbow criteria (Damaraju et al., 2014).

We investigated the temporal properties of DFC states by calculating the fractional windows (the number of total windows belonging to a given state), mean dwell time (the number of consecutive windows belonging to a given state), and the number of transitions (the number of transitions between each pair of states) (Fiorenzato et al., 2019). The differences in dynamic properties were computed by two-sample *t*-tests, except for the state distribution compared by the chi-square test.

#### Graph Theory Analysis

Graph theory parameters were analyzed using GRETNA software (http://www.nitrc.org/projects/gretna) based on the 33 independent components obtained in the ICA (Wang et al., 2015). The sparsity value of 0.34 was selected to maximize global and local efficiency (Achard and Bullmore, 2007). The global network metrics measured were global efficiency (the efficiency of parallel information transfer in a network) and the clustering coefficient (the mean of clustering coefficients of each node in a network) (Wang et al., 2011). The nodal network metrics measured were clustering coefficients (the likelihood that the neighborhoods of a given node are connected), shortest path (the mean distance between a given node and all the other nodes in the network), local efficiency (how efficient the communication is among the first neighbors of a given node when it is removed), and degree centrality (the information communication ability of a given node in the functional network) (Wang et al., 2011). The group differences in graph theory parameters were compared with two-sample *t*-tests with false discovery rate (FDR) correction.

#### Apolipoprotein E Genotyping

DNA extraction from 300 μL of whole blood per subject was performed using an SK2884 DNA extraction kit (Sangon Biotech, Shanghai, China). Apolipoprotein E (APOE) single nucleotide polymorphism (SNP) genotyping was performed for rs429358 and rs7412 using polymerase chain reaction (PCR) technology. We determined the APOE ε4 status for 50 of the 65 participants (22/33 of the NC group and 28/32 of the SCD group).

### Statistical Analysis

Age, years of education, and cognitive scores were compared using two-sample *t*-tests, while gender and APOE ε4 status were calculated by chi-square tests. We further calculated the Pearson's correlations between the altered DFC temporal properties, graph theory parameters, and cognitive measures, adjusting for age, gender, and years of education. Statistical analyses were performed with SPSS version 21.0, and *p* < 0.05 was set as the threshold for statistical significance.

## Results

### Demographic and Cognitive Characteristics

No significant differences in terms of age, gender, or years of education were found between the SCD and NC groups. The SCD participants showed abilities comparable to the controls in the global cognition, episodic memory, language, and visuospatial domains. The SCD group performed worse on the TMT-B [*t*_(63)_ = 2.624, *p* = 0.011] and SDMT [*t*_(63)_ = −2.075, *p* = 0.042]. Detailed demographic and clinical information is shown in Table 1.

**Table 1:** Demographic and clinical data.

	**NC (*n* = 33)**	**SCD (*n* = 32)**	**Statistics**	***p***
Age	64.55 ± 5.33	65.22 ± 5.02	*t*_(63)_ = 0.524	0.602
Gender (M/F)	8/25	5/27	*χ^2^*_(1)_ = 0.754	0.385
Education years	12.97 ± 3.34	12.25 ± 2.62	*t*_(63)_ = −0.965	0.338
MMSE	28.97 ± 1.31	28.66 ± 1.31	*t*_(63)_ = −0.964	0.339
AVLT immediate	17.55 ± 4.57	16.94 ± 4.77	*t*_(63)_ = −0.525	0.601
AVLT short delayed	5.27 ± 2.79	4.78 ± 2.32	*t*_(63)_ = −0.771	0.444
AVLT long delayed	5.00 ± 2.86	4.56 ± 2.38	*t*_(63)_ = −0.669	0.506
AVLT cued recall	4.70 ± 2.32	4.53 ± 2.05	*t*_(63)_ = −0.305	0.762
AVLT recognition	21.91 ± 1.44	21.50 ± 1.34	*t*_(63)_ = −1.181	0.242
AFT	19.18 ± 4.00	18.38 ± 4.80	*t*_(63)_ = −0.737	0.464
BNT	27.39 ± 2.45	27.03 ± 2.63	*t*_(63)_ = −0.575	0.567
TMT_A	58.24 ± 21.32	60.44 ± 16.66	*t*_(63)_ = 0.462	0.646
TMT_B	131.42 ± 29.66	164.06 ± 64.81	*t*_(63)_ = 2.624	0.011^*^
SDMT	41.94 ± 9.22	36.84 ± 10.55	*t*_(63)_ = −2.075	0.042^*^
CDT	27.91 ± 1.99	26.75 ± 3.04	*t*_(63)_ = −1.825	0.073
APOE (ε3ε3/ε3ε4)	17/5	23/5	*χ^2^*_(1)_= 0.183	0.669^a^

*Values are the mean ± standard deviation. MMSE, mini-mental state examination; AVLT, auditory verbal learning test; AFT, animal fluency test; BNT, Boston naming test; TMT-A, trail making test part A; TMT-B, trail making test part B; SDMT, symbol digit modalities test; CDT, clock drawing test; APOE, apolipoprotein E*.

* *p < 0.05*,

a *APOE ε4 status not determined for the whole cohort*.

### Dynamic Functional Connectivity Differences

**Figure 2:**
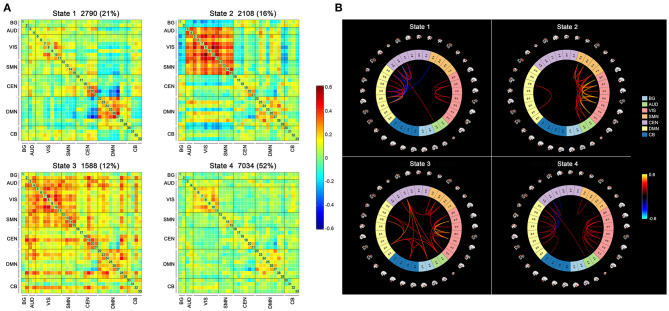
The four states identified by k-means clustering analysis and the corresponding cluster centroids. The total number and percentage of the reoccurrence times of each state are listed above each cluster **(A)**, and the 5% strongest connections of each state are shown **(B)**. BG, basal ganglia; AUD, auditory; VIS, visual; SMN, sensorimotor; CEN, cognitive executive; DMN, default mode; CB, cerebellar.

Four DFC states of the whole cohort were identified (Figure 2) as follows: (1) state 1, 21% of the windows, characterized by partly strongly connected components within the VIS, CEN and DMN, and anti-related correlations between the DMN and the other networks; (2) state 2, 16% of the windows, distinguished by the predominance of strong positive intra-network and inter-network FC in the AUD, VIS, and SMN, while negative correlations between the AUD-VIS-SMN regions and other networks; (3) state 3, 12% of the windows, a highly connected state demonstrating positive couplings of intra-network and inter-network connections involving components of nearly the whole brain; and (4) state 4, 52% of the windows, a hypo-connected state showing sparsely connected patterns located mostly within each network and between each pair of networks, except for moderate FC within the VIS and DMN.

**Figure 3:**
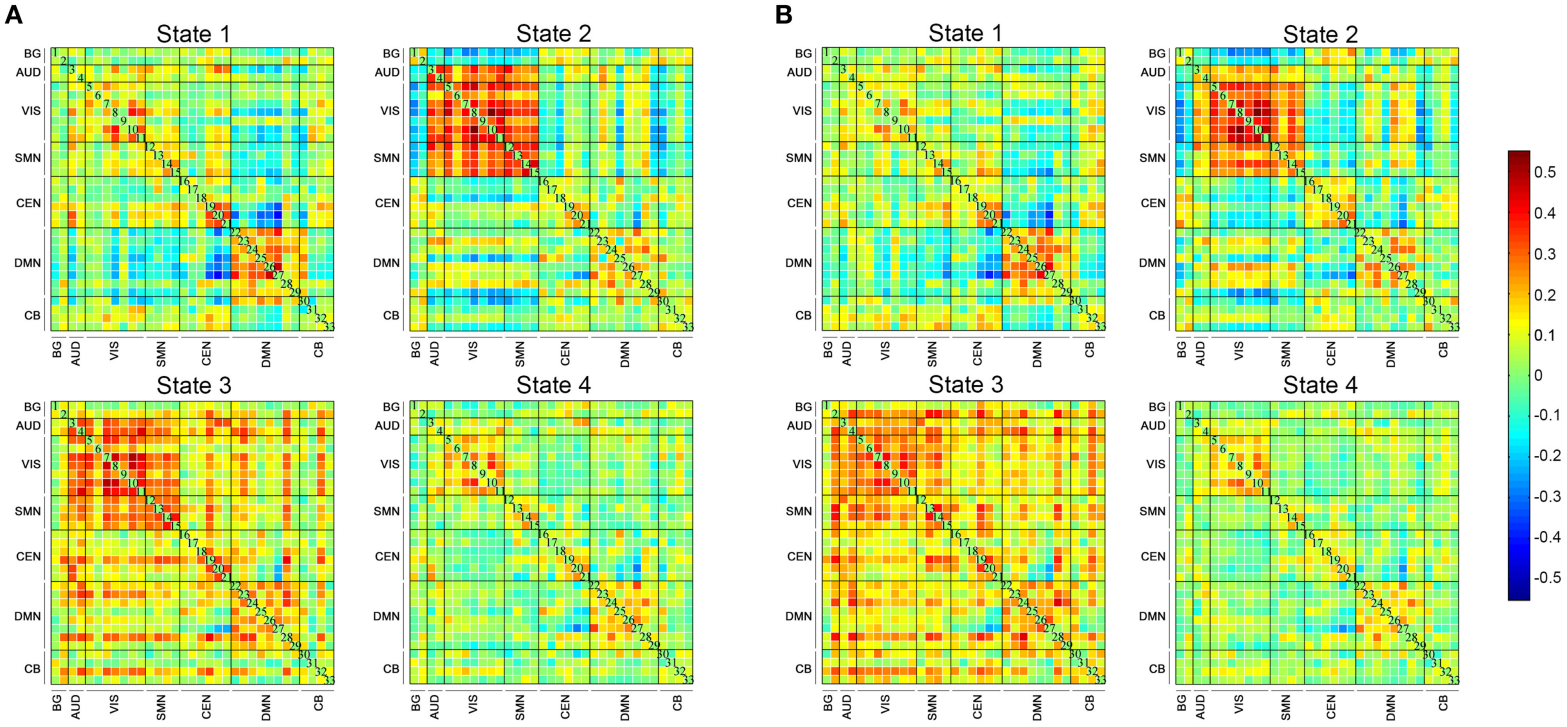
The four dynamic functional connectivity patterns of the two groups. **(A)** The centroid matrices for the normal controls. **(B)** The centroid matrices for the subjective cognitive decline participants.

**Figure 4:**
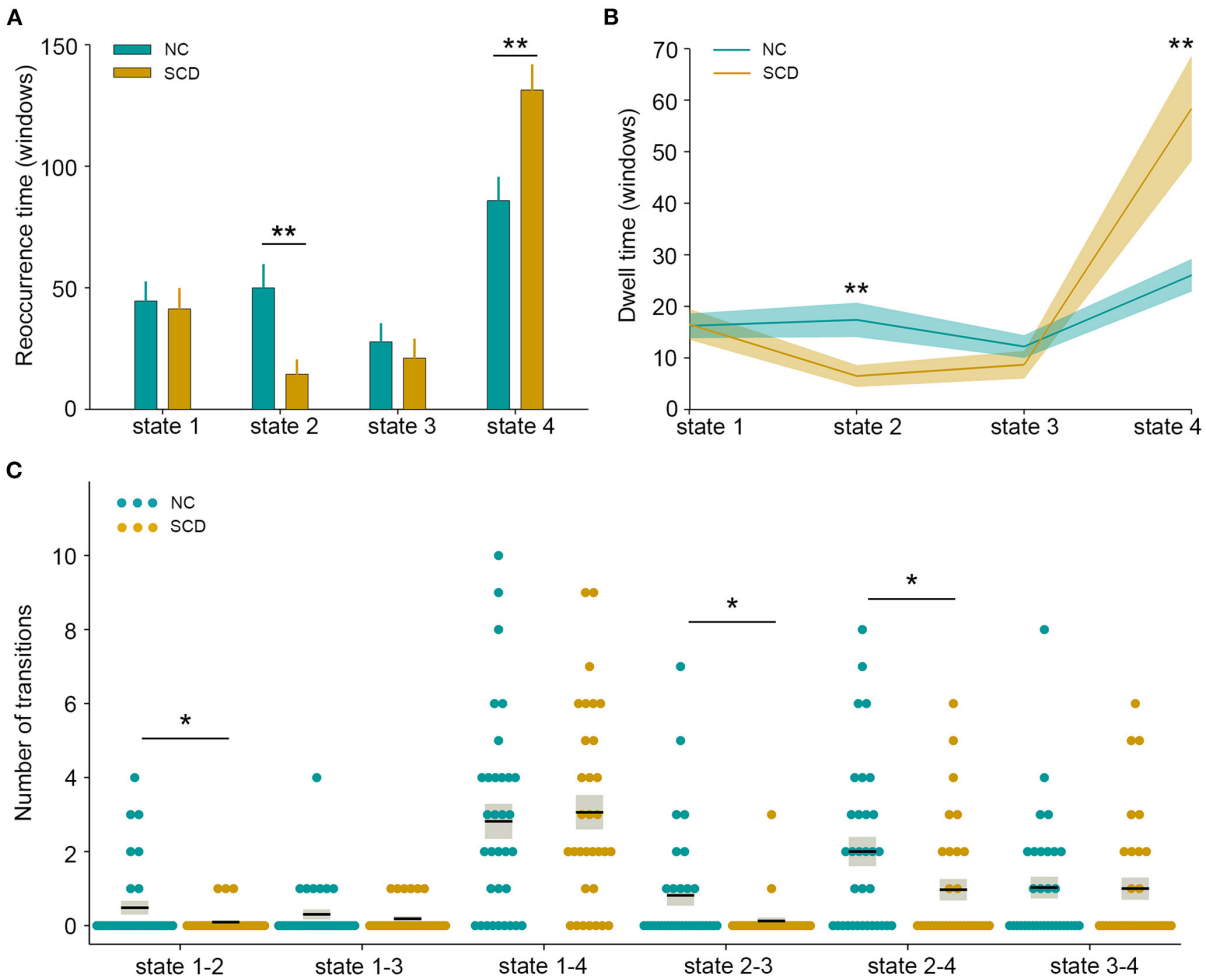
Temporal properties of dynamic functional connectivity states between the two groups. **(A)** Fractional windows in each state. **(B)** Mean dwell time in each state. **(C)** The number of transitions between pairs of states. The parameters of each individual in the normal control (NC) and subjective cognitive decline (SCD) groups are presented in blue and khaki dots respectively. The black lines indicate the mean values, and the light gray rectangles cover the data within one standard error above and below the mean. *p < 0.05; **p < 0.01.

The state- and group-specific centroids of clusters for the NC and SCD groups are shown in Figures 3A,B, respectively. The proportion of the state differed significantly between the two groups [χ^2^_(3)_ = 973.444, *p* < 0.001]. More specifically, in the SCD group, state 1 occurred slightly less frequently than it did in the NC group (19.85 vs. 21.40%) as did state 3 (10.10 vs. 13.35%). Also, state 2 occurred less frequently (6.94 vs. 23.98%), whereas state 4 occurred more often (63.12 vs. 41.27%) in the SCD group compared to the NC group. Regarding the temporal properties (Figure 4A), the SCD group was observed to have significantly reduced fractional windows in state 2 [*t*_(63)_ = −3.053, *p* = 0.003], and increased fractional windows in state 4 [*t*_(63)_ = 3.153, *p* = 0.002]. The SCD group also showed a significantly reduced mean dwell time in state 2 [*t*_(63)_ = −2.736, *p* = 0.008] and an increased mean dwell time in state 4 [*t*_(63)_ = 3.079, *p* = 0.003] (Figure 4B). Additionally, significant reductions in the transitions between state 1 and state 2 [*t*_(63)_ = −2.005, *p* = 0.049], between state 2 and state 3 [*t*_(63)_ = −2.307, *p* = 0.024], and between state 2 and state 4 were observed in the SCD group compared to the NC group [*t*_(63)_ = −2.099, *p* = 0.040] (Table 2 and Figure 4C).

**Table 2:** Dynamic functional connectivity temporal properties.

		**NC** ** (*n* = 33)**	**SCD** ** (*n* = 32)**	**Statistics**	***p***	**Cohen's d**
Fractional windows	State 1	44.52 ± 46.40	41.28 ± 48.41	*t*_(63)_ = −0.275	0.784	0.068
	State 2	49.88 ± 56.26	14.44 ± 34.37	*t*_(63)_ = −3.053	0.003^*^	0.760
	State 3	27.76 ± 43.88	21.00 ± 45.64	*t*_(63)_ = −0.609	0.545	0.151
	State 4	85.85 ± 56.06	131.28 ± 60.10	*t*_(63)_ = 3.153	0.002^*^	0.782
Fractional windows (%)	State 1	1469 (21.40)	1321 (19.85)			
	State 2	1646 (23.98)	462 (6.94)			
	State 3	916 (13.35)	672 (10.10)	*χ^2^*_(3)_ = 973.444	<0.001^*^	
	State 4	2833 (41.27)	4201 (63.12)			
Dwell time (windows)	State 1	16.22 ± 13.72	16.52 ± 16.93	*t*_(63)_ = 0.078	0.938	0.019
	State 2	17.38 ± 19.17	6.49 ± 11.97	*t*_(63)_ = −2.736	0.008^*^	0.681
	State 3	12.21 ± 12.77	8.68 ± 15.14	*t*_(63)_ = −1.015	0.314	0.252
	State 4	26.04 ± 18.19	58.41 ± 57.52	*t*_(63)_ = 3.079	0.003^*^	0.759
Number of transitions	State 1-2	0.48 ± 1.06	0.09 ± 0.30	*t*_(63)_ = −2.005	0.049^*^	0.501
	State 1-3	0.30 ± 0.77	0.19 ± 0.40	*t*_(63)_ = −0.757	0.452	0.179
	State 1-4	2.82 ± 2.71	3.06 ± 2.63	*t*_(63)_ = 0.369	0.713	0.090
	State 2-3	0.82 ± 1.61	0.13 ± 0.55	*t*_(63)_ = −2.307	0.024^*^	0.574
	State 2-4	2.00 ± 2.26	0.97 ± 1.64	*t*_(63)_ = −2.099	0.040^*^	0.522
	State 3-4	1.03 ± 1.69	1.00 ± 1.70	*t*_(63)_ = −0.072	0.943	0.018

*Values are the mean ± standard deviation*.

* *p < 0.05*.

### Graph Topological Parameters

After FDR correction, we observed no significant differences either in the global or in the nodal network metrics between the NC and SCD groups.

### Relationships Between Altered Neuroimaging Measures and Cognitive Function

Significant associations between altered neuroimaging measures and cognitive variables are summarized in Supplementary Table 1. In the whole cohort, the number of fractional windows and mean dwell time of state 4 both showed significant positive correlations with the time spent on the TMT-A (*r* = 0.343, *p* = 0.006; *r* = 0.255, *p* = 0.045, respectively). The transitions between state 1 and state 2 showed positive correlations with AVLT immediate memory scores (*r* = 0.265, *p* = 0.037), and the transitions between state 2 and state 3 were positively correlated with AVLT recognition scores (*r* = 0.257, *p* = 0.044).

In the NC group, both more fractional windows of state 4 and longer time dwelt in state 4 correlated with lower MMSE scores (*r* = −0.499, *p* = 0.005; *r* = −0.420, *p* = 0.021). The transitions between state 1 and state 2 showed positive correlations with AVLT immediate memory scores (*r* = 0.410, *p* = 0.025) and BNT scores (*r* = 0.364, *p* = 0.048).

In the SCD group, more fractional windows in state 4 were associated with longer time spent on the TMT-A (*r* = 0.370, *p* = 0.048), whereas longer time dwelt in state 2 predicted higher AVLT recognition scores (*r* = 0.392, *p* = 0.036). The transitions between state 1 and state 3, and between state 2 and state 4 were positively correlated with the AVLT recognition scores (*r* = 0.409, *p* = 0.028; *r* = 0.376, *p* = 0.045).

## Discussion

In the present study, we combined the ICA, DFC, and graph theory approaches to investigate the dynamic characteristics and global/local network topology of intrinsic connectivity networks in SCD individuals. The results revealed altered DFC temporal properties of fractional windows, mean dwell time, and the number of transitions in SCD subjects, which showed significant associations with cognitive performance. No significant differences in static parameters of graph theory were observed. These findings shed light on the role of DFC in the early detection of subjects with potential AD, and the alterations in DFC may suggest the neural basis underlying early cognitive decline.

As noted above, four distinct connectivity configurations were identified across the entire cohort. Consistent with previous findings (Allen et al., 2014; Kim et al., 2017; Viviano et al., 2017; Schumacher et al., 2019; Gu et al., 2020), the hypo-connected state occurred most frequently, that is, state 4 in the present study, characterized by a sparse connectivity pattern with relatively weak connections and the absence of strong correlations. This state profile was considered the baseline connectivity pattern, while other states with strong positive or negative connections may reflect neuropsychological processes (Viviano et al., 2017). The high occurrence of state 4 may indicate that, on the whole, the human brain prefers to be in a state with less information transfer but a more energy reservation pattern (Gu et al., 2020). In comparison with the NC group, state 2, showing hyperconnectivity within and between the AUD, VIS, and SMN, occurred 17.04% less frequently in the SCD group. In contrast, state 4 occurred 21.85% more often in SCD participants than in the NCs. The differences in state distribution suggested that the SCD group was more inclined to be in a state with reduced intra-network and inter-network interaction rather than that dominated by high AUD-VIS-SMN communication.

Variability in temporal properties of brain states during the time of the experimentally unconstrained scanning session was detected. The SCD group showed significantly fewer fractional windows and shorter mean dwell time in state 2 than the NC group, suggesting decreased within-network connectivity and reduced AUD-VIS-SMN network integration in the SCD stage. Increasing evidence has suggested that auditory, visual, and sensorimotor dysfunctions are commonly involved during AD progression and may precede the onset of cognitive impairments and dementia (Albers et al., 2015; Deng et al., 2016). Our results of weak connectivity in sensory domains may provide an explanation for these deficits in the earliest stages of AD. In addition, the reduced interaction among AUD-VIS-SMN networks was consistent with the concept that cognitive decline in AD is a disconnection syndrome closely associated with the functional segregation of coordinated brain networks (Delbeuck et al., 2003). A recent DFC study has shown significantly lower temporal variability involving the regions of the SMN and VIS in AD dementia patients, which could be related to reduced flexibility in sensory, motor, and visual functions (Gu et al., 2020). Another study has observed a significant reduction in the frequency and mean dwell time in the state characterized by strong positive correlations within and between the visual and motor networks in AD dementia patients (Schumacher et al., 2019). The present study extends previous findings by showing that brain network reorganization in SCD individuals presents a similar pattern to that of AD dementia patients.

The SCD group also showed significantly increased fractional windows and a significant increase in mean dwell time in state 4 compared to the NC group. A previous study has observed that AD dementia patients spent more time than NCs in sparse connectivity configurations, indicating their inability to switch out of states with low inter-network connectivity into more highly and specifically connected network configurations; this deficiency might be related to cognitive deterioration (Schumacher et al., 2019). Our results of more time spent in the sparsely connected state in the SCD group supported the concept that SCD was a preclinical stage of the AD spectrum from the perspective of DFC state patterns. Notably, the SCD group showed fractional windows and a mean dwell time in state 3 similar to those of NCs, which was dominated by strong connections within and between distinct functional networks. The SCD group also showed similar fractional windows and a similar mean dwell time in state 1, which was characterized by anti-correlations between the DMN and other networks; these anti-correlations have been shown to be crucial for cognitive processes (Fox et al., 2005; Baggio et al., 2015). The absence of this antithetic association has been reported in MCI and AD dementia patients (Esposito et al., 2018; Schumacher et al., 2019). We speculated that contrary to the symptomatic AD stage, the strong connections in the whole brain networks and the antagonism between the DMN and task-positive networks may remain stable in the SCD stage to support objectively unimpaired cognition, and this speculation remains to be further validated.

Regarding the number of transitions between distinct states, the SCD group demonstrated significantly reduced transitions between state 1 and state 2, between state 2 and state 3, and between state 2 and state 4 in the present study. State transitions are believed to reflect neural metastability, which enables multiple brain regions to engage and disengage flexibly in coordination without being locked into fixed interaction patterns (Li et al., 2017). Frequent transitions between discrete connectivity patterns also facilitate flexible information integration and intensive information exchange across multiple specialized subnetworks (Li et al., 2017). The configurations of multiple brain regions interacting in complex and flexible communication patterns may be disrupted in SCD individuals. These results elucidated the vulnerability of rs-fMRI networks in the SCD stage and emphasized the importance of investigating the dynamic characteristics of the brain.

We observed significant associations between DFC properties and cognitive performance. The more time participants spent in state 4, the worse executive and general cognitive function they had. State 4 represents the most hypo-connected networks among all 4 states, including weak intra-network connectivity in the CEN and weak inter-network connectivity between the CEN and other brain modules, which may contribute to ineffective information transfer and processing, thus resulting in worse executive and general cognitive ability. The more frequent transitions between states predicted better performance on immediate and recognition memory tests, which may imply potential relationships between neural flexibility and memory function. A previous study has revealed a reduction in brain metastability related to cognitive impairments in cognitive flexibility, speed of information processing, and associative memory (Hellyer et al., 2015). The inflexibility of functional networks may result in the loss of memory encoding and retrieval efficiency in SCD individuals. We also observed a significant association between longer time dwelt in state 2 and better recognition memory performance in the SCD group, indicating that functional integration of the AUD-VIS-SMN may help strengthen memory function. In addition, increased switches between distinct dynamic FC states may also contribute to better language ability. These findings provide evidence that altered dynamic functional brain organization is linked to cognitive function, which may further serve as the neural substrates underlying cognitive decline in the SCD stage. Notably, the relationships between DFC temporal properties and the cognitive variables reported above did not survive multiple comparison corrections and further research is needed to confirm these exploratory results.

In contrast to the remarkable dynamic FC alterations, we did not find differences in either global or local topological parameters by graph theory approaches. Previous studies have shown topological alterations in SCD subjects (Chen et al., 2020; Xu et al., 2020), and we speculated that the discrepancies may be attributable to the different diagnostic criteria for SCD, the variations in demographics of the cohorts, and methodological aspects (Wang et al., 2020). These studies also revealed no group differences in the static analysis of global and local efficiency between AD dementia patients and NCs (Peraza et al., 2015; Schumacher et al., 2019). Our findings provide further evidence that DFC, which captures the temporal variations of FC, may be a more informative representation of functional brain networks than SFC for the preclinical detection of incipient AD patients.

Several limitations in the present work should be considered. First, this is a cross-sectional study conducted in a small cohort, while AD is a progressive neurodegenerative disorder; therefore, longitudinal studies with large cohorts are needed to elucidate the role of DFC in the whole AD spectrum. Second, the acquisition time of rs-fMRI data was 8 min 7 s, though researchers have suggested that DFC analysis should be performed with rs-fMRI acquisition times > 10 min. Third, no pathological evidence from amyloid or tau positron emission tomography (PET) and CSF was available. The impact of AD pathology on the interaction and modulation of brain functional networks needs to be further investigated. Notably, the *p* values in the correlational analysis may not remain significant if multiple comparison corrections were applied, and the large number of zero values may have an impact on the results; thus, the associations between altered DFC parameters and cognitive variables were exploratory results and warrant further validation. Furthermore, the APOE ε4 genotype may have an impact on the fMRI measures, thus in our future study with a larger sample size of APOE ε4 carriers, we will investigate differences in DFC properties between APOE ε4 carriers and non-carriers in SCD subjects.

## Conclusion

In the present study, we investigated alternations in DFC temporal properties in SCD individuals, with a focus on the fractional windows, mean dwell time, and state transitions. We observed increased fractional windows and mean dwell time in a hypo-connected state and a reduced number of state transitions in the SCD group compared to the NC group. Furthermore, the altered DFC measures were significantly correlated with cognitive variables. Our findings suggested that DFC analysis may provide novel insights into the organization principles of brain networks underlying early cognitive decline in the SCD stage and benefit the preclinical detection of incipient AD patients.

## Data Availability Statement

The raw data supporting the conclusions of this article will be made available by the authors, without undue reservation.

## Ethics Statement

The studies involving human participants were reviewed and approved by Human Participants Ethics Committee of the Nanjing Drum Tower Hospital. The patients/participants provided their written informed consent to participate in this study.

## Author Contributions

QC was responsible for the conception and design of the present study, execution of the experimental work, and wrote the first draft of the manuscript. JL and XZ organized the research project and reviewed and critiqued the manuscript. YS, WC, and XL executed the experimental work. WZ and ZQ reviewed and critiqued the statistical analysis. BZ guided the design of the study protocol, and reviewed and critiqued the manuscript. All authors contributed to the article and approved the submitted version.

## Conflict of Interest

The authors declare that the research was conducted in the absence of any commercial or financial relationships that could be construed as a potential conflict of interest.
