# Supplementary material for: Alterations in Dynamic Functional Connectivity in Individuals With Subjective Cognitive Decline
Source: Front Aging Neurosci. 2021 Feb 3;13:646017. doi: 10.3389/fnagi.2021.646017 (PMC7886811; doi:10.3389/fnagi.2021.646017)
Supplement: Supplementary file 1 [file Data_Sheet_1.docx]

Supplementary Table 1 Significant correlations between dynamic functional connectivity temporal properties and cognitive data

|  |  |  | Total sample | | |  | NC group | | |  | SCD group | |
| --- | --- | --- | --- | --- | --- | --- | --- | --- | --- | --- | --- | --- |
|  |  |  | AVLT  immediate | AVLT  recognition | TMT-A |  | MMSE | AVLT immediate | BNT |  | AVLT  recognition | TMT-A |
| Fractional windows | state 2 | r | * | * | * |  | * | * | * |  | * | * |
|  |  | p | * | * | * |  | * | * | * |  | * | * |
|  | state 4 | r | * | * | 0.343 |  | -0.499 | * | * |  | * | 0.370 |
|  |  | p | * | * | 0.006 |  | 0.005 | * | * |  | * | 0.048 |
| Dwell time | state 2 | r | * | * | * |  | * | * | * |  | 0.392 | * |
|  |  | p | * | * | * |  | * | * | * |  | 0.036 | * |
|  | state 4 | r | * | * | 0.255 |  | -0.420 | * | * |  | * | * |
|  |  | p | * | * | 0.045 |  | 0.021 | * | * |  | * | * |
| Number of Transitions | state 1-2 | r | 0.265 | * | * |  | * | 0.410 | 0.364 |  | 0.409 | * |
|  |  | p | 0.037 | * | * |  | * | 0.025 | 0.048 |  | 0.028 | * |
|  | state 2-3 | r | * | 0.257 | * |  | * | * | * |  | * | * |
|  |  | p | * | 0.044 | * |  | * | * | * |  | * | * |
|  | state 2-4 | r | * | * | * |  | * | * | * |  | 0.376 | * |
|  |  | p | * | * | * |  | * | * | * |  | 0.045 | * |

P values were adjusted for age, gender, and years of education. MMSE: mini-mental state examination; AVLT: auditory verbal learning test; TMT-A: trail making test part A; BNT: Boston naming test.
